# Insect–Virus Interactions: A Fascinating Area of Research That Requires Ongoing Attention

**DOI:** 10.3390/v16111672

**Published:** 2024-10-25

**Authors:** Li-Long Pan

**Affiliations:** 1 Ministry of Agriculture Key Lab of Molecular Biology of Crop Pathogens and Insects, Zhejiang Key Laboratory of Biology and Ecological Regulation of Crop Pathogens and Insects, Institute of Insect Sciences, Zhejiang University, Hangzhou 310058, China; panlilong@zju.edu.cn; 2 The Rural Development Academy, Zhejiang University, Hangzhou 310058, China

As the most abundant and diverse groups of animals, insects play many important roles in the ecosystem, such as those of herbivores, vectors, and pollinators. In the life cycle of these small creatures, insects are constantly associated with microorganisms, including viruses. First, insects may serve as vectors of plant viral pathogens that dictate viral spread and epidemics [1]. Second, insects may be infected by entomopathogenic viruses, leading to drastic changes in insect physiology and biochemistry [2]. Third, insects are ubiquitously infected by insect-specific viromes [3]. These insect–virus interactions serve as important determinants of the distribution and abundance of the two kinds of organism in natural and artificial ecosystems. Therefore, understanding these interactions is imperative, especially in the era of global change when pest predication and management become increasingly difficult.

Insects, especially hemipteran ones such as aphids and whiteflies, are responsible for the dissemination of the majority of known plant viruses [1]. In recent decades, whitefly-borne viruses and viral diseases have gradually emerged as the major threats in the production of many crops [4]. To understand the status of whitefly and whitefly-borne viral diseases, among other pests, Wosula et al. conducted extensive field surveys in 2022 and 2023 and determined the prevalence, incidence, and severity of cassava mosaic disease and cassava brown streak disease, and the abundance of whitefly in six counties of western Kenya. Their results demonstrate that whiteflies and whitefly-borne cassava viral diseases continue to be important constraints to cassava production in western Kenya [5]. Amid the transmission of plant viruses by whitefly, other plant-feeding insects may modulate plant physiology and potentially the resistance against the vector or the virus and virus transmission efficiency. To clarify this issue, Legarrea et al. explored the impact of the mirid bug *Dicyphus hesperus* Knight on the acquisition and transmission of a begomovirus by whitefly. They found that although the mirid bug induced a defensive plant response, the acquisition and transmission of begomovirus was not significantly affected [6]. For the management of whitefly-borne begomoviruses, one of the strategies is to interfere with whitefly-mediated virus transmission. In this regard, Wei et al. explored the use of a plant-derived volatile component, d-limonene, in managing whitefly’s transmission of a begomovirus. They showed that d-limonene impeded whitefly feeding and significantly reduced virus acquisition and transmission by whitefly [7].

Entomopathogenic viruses hold promise for controlling insect pests in an environmentally friendly way, and thus their interaction with insect hosts has been extensively characterized [2]. Upon infection, entomopathogenic viruses may interact with each other, leading to an altered infection of the involved viruses and host phenotype. To decipher the homologous superinfection exclusion induced by a baculovirus, Fu et al. characterized the cell cycle and its regulators. They found that the latent infection of baculoviruses in G1 phase resulted in G2/M arrest, thereby alleviating the homologous superinfection exclusion [8]. Furthermore, Durand et al. summarize the interactions among bee-infecting viruses. They first summarize the current knowledge of bee antiviral immunity, and then the interactions among entomopathogenic viruses and their interactions with insect hosts. Finally, they put forward novel hypotheses and suggest future research directions [9]. The fruitfly, *Drosophila melanogaster*, is a widely used model in exploring the topic of insect antiviral immunity. Using this model system, Wang et al. explored the contribution of nicotinic acetylcholine receptor alpha6 in defenses against sigmavirus. They found that nicotinic acetylcholine receptor alpha6 positively regulated fly antiviral immunity by activating the immune deficiency (IMD) pathway [10].

Investigations in the last few years have uncovered the ubiquitous presence of insect-specific viruses in many insect linages [3]. These viruses may significantly modulate the life history of their insect hosts, and thus spark extensive research interest in the scientific community. To expand the knowledge of insect-specific viromes, Thekke-Veetil et al. and Caldas-Garcia et al. profiled virus diversity in a potato leafhopper and six parasitoid wasps, respectively. Thekke-Veetil et al. identified eight putative insect-infecting viruses, one of which was novel. Genomic analyses of the novel genome sequences and their evolutionary relationships with related family members were also conducted [11]. Caldas-Garcia et al. identified 18 viruses from 10 families, of which 16 were described for the first time. Additionally, they found evidence of inter-species transmission of an insect-specific virus [12].

We are grateful to all authors for their timely contributions. In closing, while some new knowledge has been provided in this Special Issue, further investigation is warranted. Future works may focus on the mechanisms underlying plant virus transmission by insect vectors, molecular warfare between insect hosts and entomopathogenic viruses, and functional characterization of insect-specific viruses.
